# Evaluation of the biological, physical, mechanical and chemical properties of orthodontic primer modified by nano-chitosan loaded with bioactive materials

**DOI:** 10.1016/j.jobcr.2025.03.002

**Published:** 2025-03-13

**Authors:** Lara Riyadh Al-Banaa, Ali R. Al-Khatib, Fawzi Habeeb Jabrail

**Affiliations:** a Department of Pedodontics, Orthodontic and Preventive Dentistry. College of Dentistry/Mosul University/ Iraq, Iraq; b Department of Chemistry, College of Science, University of Mosul, Mosul, Iraq

**Keywords:** Antibacterial, Adhesive, Chitosan, Gallic acid, Nanoparticles, Orthodontic, Remineralization

## Abstract

**Objective:**

The primary objective was to evaluate the antibacterial and remineralizing effects of modified orthodontic primer containing nano-chitosan loaded with bioactive materials (gallic acid and calcium phosphate). The secondary objectives were to assess the physical, mechanical, and chemical properties of the adhesive primer.

**Materials and methods:**

The chitosan loaded with gallic acid and calcium phosphate was prepared and characterized by FESEM-EDX. Then it was mixed with Transbond XT primer at two different concentrations to prepare 3 groups: Control, 5 % and 10 % Nano-Chitosan/Gallic acid + Calcium Phosphate Primer (NC/GACP). The primer groups were evaluated based on their Shear Bond Strength (SBS), Adhesive Remanent Index (ARI), Wettability, Degree of Conversion (DC), Antibacterial and Remineralization properties. Statistical analysis was conducted using one-way ANOVA and Tukey's post hoc test, all significant differences were set at P < 0.05.

**Results:**

The modified primer groups containing 5 % and 10 % NC/GACP showed a significant increase (P < 0.05) in SBS and DC compared to the control group, with no significant differences observed in wettability. The ARI scores were mainly 3 meaning all the adhesive remained on the enamel after debonding. Additionally, both modified primer groups exhibited significant antibacterial activity (P < 0.05). Furthermore, the 5 % NC/GACP primer group showed the highest calcium and phosphate weight percentages and Ca/P ratio compared to the other groups (P < 0.05).

**Conclusions:**

The orthodontic primer modified by nano-chitosan/gallic acid + calcium phosphate can be considered a novel antimicrobial and remineralizing adhesive that enhances the physical, chemical and mechanical properties of the adhesive.

## Graphical abstract

**Figure:**
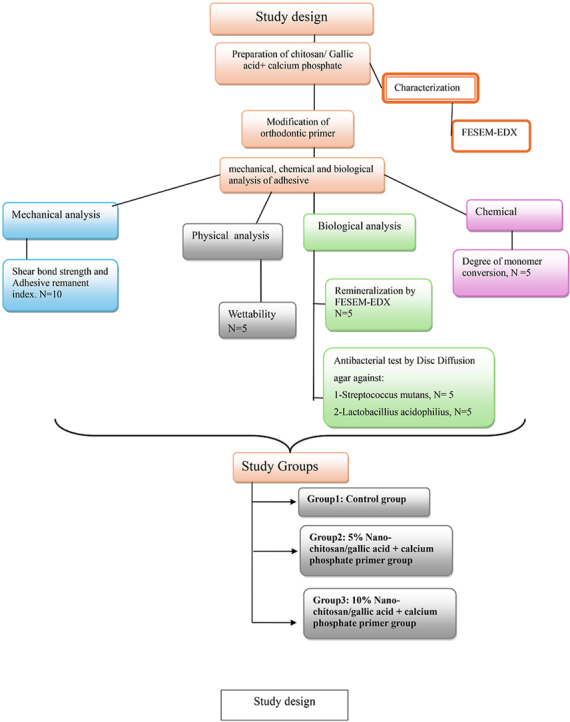

## 1 Introduction

White-Spot Lesions (WSLs) are one of the common complications of comprehensive orthodontic treatment. There are many modalities for the prevention of WSLs which include patient education on oral hygiene, the use of topical fluorides, a sugar-controlled diet, and the use of fluoride or chlorhexidine-containing dentifrices.^1^^,^^2^ Also, the use of various remineralization agents like fluoride, Calcium Carbonate (CaCO3), Casein Phospho Peptide (CPP), Amorphous Calcium Phosphate (ACP), Hydroxyapatite (HAP) have shown their success in preventing WSLs.^3^ Recently various nanomaterials are used in orthodontics as specific drug delivery systems.^4^

Gallic acid (GA) is a 3,4,5-trihydroxybenzoic acid, has the chemical formula C_6_H_2_(OH)._3_COOH. GA esters are found in numerous plants and are recognized as gallates. It is extracted from a variety of plants including Oak bark, Tea leaves, and Galla chinensis extract with a chemical structure of 3,4,5-trihydroxy benzoic acid. It has been confirmed to be a strong antibacterial agent and can affect the demineralization and remineralization of dental hard tissues by acting as a calcium ion carrier.^5^

The use of nano-chitosan as a drug carrier for calcium phosphate and/or gallic acid due to its unique properties. It has excellent biocompatibility, biodegradability, non-toxicity, and antimicrobial properties. It can form a stable matrix and encapsulate bioactive molecules or minerals. Also, it ensures a controlled and targeted delivery and enhances the effectiveness of therapeutic agents.^6^^,^^7^

Previous studies analysis showed that most research evaluated the effects of either chitosan or calcium phosphate compounds added to bonding agents or orthodontic composites, focusing on either antibacterial or remineralizing properties. However, there is a lack of research on the effectiveness of nano-chitosan loaded with these bioactive materials in preventing white spot lesions.

Thus, the present study was designed to evaluate the combined antibacterial and remineralizing effects of orthodontic primer modified with nano-chitosan loaded with gallic acid and calcium phosphate and to assess the physical, mechanical, and chemical properties of orthodontic primer.

## 2 Materials and methods

This research has been approved by the ethical committee at the College of Dentistry/University of Mosul under the no (UoM.Dent. 23/24) on May 02, 2023.

The sample size was calculated using the following formula:

N = [(4σ2) (Zα + Zβ)2] ÷E2 

where: *N*: The number of experimental samples,

*σ*: The assumed standard deviation, it was = 2.31^8^ (SBS) and, *Zα* = 1.96 for a = 0.05 (two-tailed), *Zβ* = 0.80 for the 80 % power, *E*: The detectable difference between treatment means = 4.

$$N=\left[4{\left(2.31\right)}^{2}{\left(1.96+0.80\right)}^{2}\right]÷4=10.1$$

Accordingly, the sample size estimation was N = 10 teeth for each study group, regarding the SBS test.

### 2.1 Preparation of nano-chitosan loaded with bioactive materials

To prepare a chitosan solution, 1.0 g of nano-chitosan powder (Nanochemazone, Edmonton, Alberta, Canada) was dissolved in 100 ml of deionized water, then 1 ml of 2 % (v/v) acetic acid solution (Merck KGaA, Darmstadt, Germany) was added dropwise to the chitosan solution. The mixture was stirred for 3 h at room temperature to ensure the complete dissolution of chitosan and the formation of a homogenous solution.^9^

Nano-chitosan loaded with bioactive materials was prepared by mixing 200 mg of both gallic acid (Fluka AG, Buch, Switzerland) and calcium phosphate (Thomas Baker, Mumbai, India) with a 100 ml of nano-chitosan solution at 0.2 % (w/v) ratio. The mixture was kept under magnetic stirring for 3 h at room temperature and the pH of the final solution was maintained at 7.0 by adding 0.05 M NaOH.^10^

### 2.2 Preparation of the modified orthodontic primer

The incorporation of nano- chitosan/bioactive materials solution into the Transbond XT primer was based on the mass fraction formula (mass percentage or wt./wt.). Nano-chitosan loaded with bioactive materials (calcium phosphate and gallic acid) was added to the primer at concentrations of 5 % and 10 % weight-to-weight ratios. To standardize the process, one drop of primer (50 μL) was measured using a micropipette. A sensitive weight scale determined that one drop weighed 0.05 g. Twenty drops of primer, equaling 1 g, were then mixed with 0.05 g and 0.1 g of nano-chitosan loaded with bioactive materials to prepare the 5 % and 10 % modified primers, respectively. Mixing was carried out in a semi-dark environment inside microtubes using the straight head of a dental probe until a uniform consistency was achieved. Additionally, a dental vibrator was used for 2 min to ensure better distribution of the material within the primer. The microtubes were wrapped with dark tape to prevent light exposure. The study groups are.

1. Control group: Transbond XT orthodontic primer without any modifications
2. 5 % NC/GACP: 5 % Nano-chitosan/gallic acid + calcium phosphate modified orthodontic primer.
3. 10 % NC/GACP: 10 % Nano-chitosan/gallic acid + calcium phosphate modified orthodontic primer.

### 2.3 Testing procedures

The following tests were performed for analysis of the modified orthodontic primer.

1. Characterization of Nano-Chitosan/bioactive materials solution by Field Emission Scanning Electron Microscopy (FESEM) and Energy Dispersive X-ray Spectroscopy (EDX)

The FESEM-EDX technique was used to determine the morphology, distribution and elemental composition of bioactive materials within the nano-chitosan matrix.

**Fig. 1:**
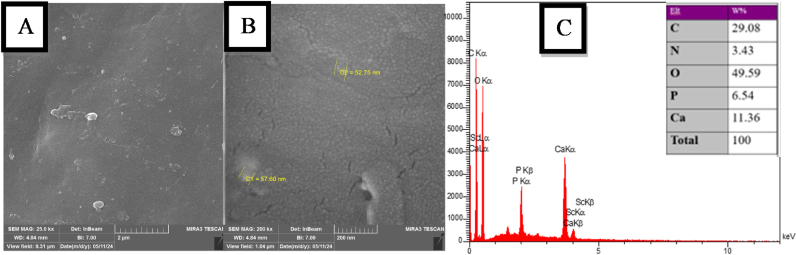
Representative FESEM image of the prepared chitosan/calcium phosphate and gallic acid, (A): The particles shapes and distribution at 25 kx (B): The particles size at 200 kx, (C): The EDX spectrum with elements percentage in weight of prepared hydrogel.

The FESEM images (Fig. 1 A) at 25 kx magnifications revealed that the prepared chitosan/calcium phosphate and gallic acid showed a homogeneous distribution of the particles within the chitosan matrix with mixed larger and smaller pale spots particles of both materials, these particles appeared as white diffused spots. In (Fig. 1B), at 200kx magnifications, the size of the particles was within a nano-size scale (50–60 nm). The EDX of chitosan/calcium phosphate + gallic acid (Fig. 1 C) represented a higher proportion of Carbon (C) and Oxygen(O) with small percentage of Nitrogen (N), Calcium (Ca) and Phosphate (P).

## 3 Shear bond strength (SBS)

Thirty maxillary first premolar extracted teeth for orthodontic purposes were used in this study. The teeth were examined thoroughly without caries, filling, or enamel cracks. After removing the soft tissue remnants and washing them in tap water, the teeth were stored in 0.1 % thymol.^11^ The teeth were mounted in plastic rings using cold cure acrylic resin to the level of the cementoenamel junction. A surveyor was used to orient the buccal surface of the tooth parallel to the surveyor rod and perpendicular to the base of the mold. Three groups of teeth were randomly selected and divided into: Control unmodified group, 5 % and 10 % NC/GACP modified primer groups. After polishing the buccal surfaces of the teeth with non-fluoridated pumice, they were rinsed with water and dried. For brackets bonding, 37 % phosphoric acid was used to etch the teeth for 20 s, rinsed for 10 s, and air dried. The unmodified and modified primer was painted on the premolar buccal surfaces, and a gentle airflow was applied to disperse excess primer. The orthodontic brackets (Stainless Steel Metallic Brackets, Standard Edgewise type, 10 mm^2^ bracket base surface area, Dentaurum, Germany) were adapted using a bracket gauge about 4.5 mm from cusp tip to bracket slot and pressed by a load of 200 g for standardization. After removal of the extra adhesive, the brackets were cured from both the mesial and distal side for 40 s, using an LED light curing device 1500 mw/cm^2^, keeping 2 mm away from the bracket. The SBS test was measured in the universal testing machine (Gester Instrument Co, Fujian, PR China), by positioning the knife-edge chisel at the tooth-bracket interface directed in an occlusal-gingival direction. The speed of the crosshead was adjusted to 0.5 mm/min, the results of the fracture force were electronically recorded in Newton, and they were converted to Megapascals by dividing the force by the surface area of the bracket base.^12^

## 4 Adhesive remnant index (ARI)

After the brackets were debonded in SBS testing, the enamel surface of all teeth was examined under a stereomicroscope (Optika, Italy) at 10× magnification. Digital images were captured to evaluate the amount of adhesive resin remaining on the tooth. The ARI scores followed the method described by Artun & Bergland,^13^ which entails the following categories.

• 0: No adhesive left on the tooth
• 1: Less than half of the adhesive left on the tooth
• 2: More than half of the adhesive left on the tooth
• 3: The entire adhesive left on the tooth, with a distinct impression of the bracket mesh.

## 5 Testing the wettability, contact angle measurement (CA)

**Fig. 2:**
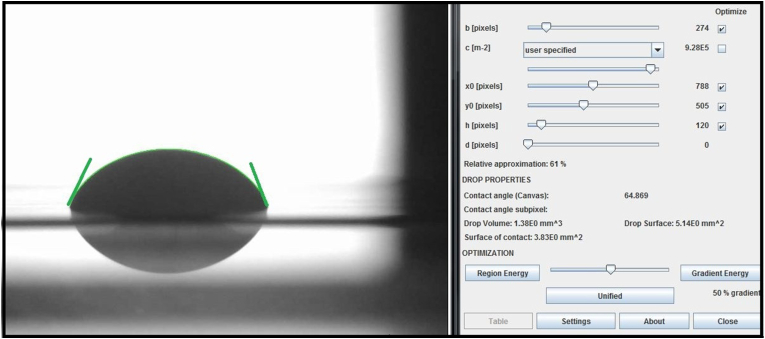
Contact angle measurement of droplet by Image J software.

Wettability was assessed by measuring the contact angle of the adhesive primer^14^ by a sessile drop method at room temperature (25 ± 0.5 °C) using a contact angle goniometer. A drop (5 μl) of each group (control primer, 5 %, and 10 % NC/GACP) was dispensed using a micropipette onto a smooth, flat aluminum plate covered with polytetrafluoroethylene tape. The plate was then positioned on the sample table of a contact angle goniometer. This procedure was repeated five times for each group, and a total of 15 images were captured. The contact angles were measured using drop shape analysis in the Image J software program (Fig. 2).

## 6 Degree of monomer conversion (DC)

The degree of conversion of control and modified adhesive resin was measured using a Fourier transform infrared spectrometer equipped with 45-degree angle attenuated total reflectance (ATR) accessory. The spectrum was carried out in the absorbance mode with a wavelength range 400–4000 cm^−1^, 24 scans at 4 cm−1 resolution. The uncured and cured specimens were placed on the diamond of the metal circlip of the FTIR.

The DC% was measured by a relative percentage basis (the tangent baseline and the two-frequency method) employing the C═C aliphatic bond stretching vibrations (analytical bond at 1638 cm-1) and the C..C aromatic bond stretching vibrations (reference bond at 1608 cm-1) of the polymerized and unpolymerized samples.^15^ In this study, the degree of conversion was calculated based on the peak area under the curve obtained from the tangent baseline connecting the trough of two peaks using Origin software. The following equation was used to determine the DC:

%DC=1–((a/b)/(c/d))∗100

DC = degree of conversion, a = peak area at 1638 after curing, b = peak area at 1607 after curing, c = peak area at 1638 before curing and d = peak area at 1607 before curing.

## 7 Antibacterial assessment: disc agar diffusion (DAD)

The clinically isolated bacterial strains were obtained from the College of Dentistry/Mosul University. The antibacterial properties of the modified primer were tested against *Streptococcus mutans* (gram-positive bacteria) and *Lactobacillus acidophilus* (gram-negative bacteria) through the release and diffusion of chitosan and gallic acid bioactive materials from disc samples by disc agar diffusion assay (Kirby-Bauer method).^16^

Plastic molds (6 mm in diameter and 2 mm in thickness) were used to fabricate disks for all groups (total discs = 15, n = 5 for each group). The primer was poured into the mold and light-cured for (10) sec. At the top and (10) sec. At the bottom of the mold, after curing, the discs were sterilized for 30 min With 70 % alcohol at room temperature.

*The Streptococcus mutans* and *lactobacillus acidophilus* were reactivated from their stock culture by regrowth in BHI broth. After 24 h, A sterile wire loop was used to smear the bacterial culture on the Mueller-Hinton Agar plate, and the agar surface was dried for about (5) min. The disks were gently pressed on the agar by sterilized forceps. Plates incubations were done in anaerobic conditions at 37 °C for 48 h. The bacterial inhibition zone was measured in millimeters around the disks. Five Petri dishes were used for each type of bacteria. Each Petri dish consisted of 3 disks: control, 5 % NC/GACP and 10 % NC/GACP.

## 8 FESEM-EDX analysis of remineralization

The study samples consist of 15 human upper premolar teeth (n = 5) with normal shape and size, recently extracted for orthodontic treatment purposes. The buccal surfaces of teeth were free of enamel cracks, fractures, filling, caries, abrasion, and hypoplastic enamel.

The buccal surfaces of upper premolars crowns of all groups were subjected to demineralization to eliminate false positive results of remineralization. First, they were painted with a nail varnish leaving a window of enamel about 3 × 4 mm exactly at the middle third of the buccal surface exposed to the acid attack, while most of the crown was protected by an acid-resistant varnish.^17^ Each tooth was immersed in 40 ml of acidified buffered demineralizing solution at room temperature for 96 h to develop an initial artificial carious lesion. The demineralizing solution contained (2.2) mmol/L CaCl2, (2.2) mmol/L NaH2PO4, and (50) mmol/L acetic acid. The solution was changed daily and the pH was regularly adjusted to maintain it at 4.5 b y NaOH.^18^

At the end of demineralization, a chalky white appearance was observed at the exposed window in the middle third of the buccal crown surface. A thin layer of the control and modified primer (5 and 10 % NC/GACP) were applied to the window of the teeth and light-cured for 10 s according to the manufacturer's instructions. After that, each group was placed in a separate closed container with 40 ml of artificial saliva and stored for 4 months to allow remineralization. Artificial saliva composition included^19^: (0.4 g/L) KCl, (0.4 g/L) NaCl, (0,795 g/L) CaCL_2_.2H_2_O, (0,780 g/L) NaH_2_PO_4_.2H_2_O, (0.005G/L) Na_2_S.9H_2_O and (1 g/L) Urea, (1000 ml) distilled water.

At the end of the remineralization period, the teeth were washed with distilled water. First, the crowns were sectioned from the roots approximately two mm below the cementoenamel junction and perpendicular to the long axis, the coronal part of each tooth was longitudinally sectioned from the lingual to the buccal direction in the mid of the occlusal surface of the premolars using a water-cooled, low-speed handpiece with double sided diamond disc. The cut surfaces were stored in distilled water until measurements. For FESEM-EDX analysis, all specimens were dehydrated in an ascending series of aqueous ethanol (70 %, 80 %, 90 %, 95 %, and 100 %), then mounted on aluminum stubs with a conductive double-sided adhesive carbon tape and sputter coated with gold to increase the conductivity of the sample, finally examined by FESEM at 20 kV accelerating voltage and 10 mA. For better visualization of a sample, the images were captured at different magnifications.

**Fig. 3:**
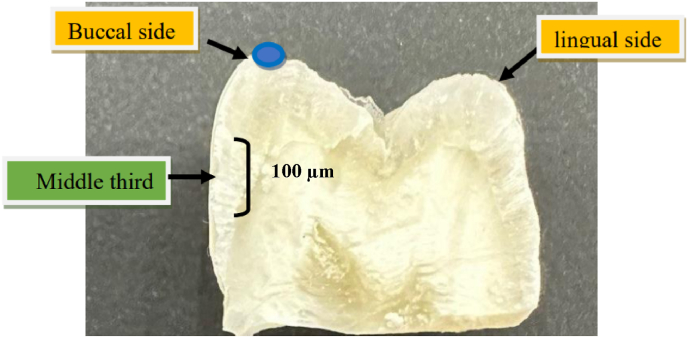
The target area of FESEM-EDX measurement of tooth cross section at 100 μm from the buccal surface.

The elemental analysis (calcium and phosphate concentration in weight percent) was performed using an EDX detector attached to the same FESEM. The cut surfaces of the teeth were examined carefully at a depth of 100 μm from the bonding surface to obtain representative photomicrographs and measurements^20^ (Fig. 3).

### 8.1 Statistical analysis

The statistical analysis was conducted using the SPSS software version 22. The Shapiro-Wilk test showed that data in SBS, CA, DC, antibacterial and EDX were normally distributed within all groups at P > 0.05. So, the results of the study were analyzed using a one-way analysis of variance (ANOVA) followed by the post-hoc Tukey's test. Statistical significance was set at P< 0.05 %.

## 9 Results

The results of SBS among three groups were presented in Table 1. The SBS mean values were 15.19 ± 2.4, 21.3 ± 3.5 and 22.6 ± 3.6 in control, 5 % NC/GACP and 10 % NC/GACP groups respectively, and the difference among them was statistically significant (P = 0.000). Compared to the control group, the SBS was significantly more in 5 % NC/GACP group (P = 0.001) and 10 % NC/GACP group (P = 0.000).

**Table-1:** Details of shear bond strength (SBS), contact angle (CA) and degree of conversion (DC) among three groups.

Tests	Groups				Post hoc Tukey test∗ p-value		
	Control	5 % NC/GACP	10 % NC/GACP	ANOVA p-value (Sig.)	Control Vs. 5 % NC/GACP	Control Vs. 10 % NC/GACP	5 % NC/GACP Vs. 10 % NC/GACP
**SBS (mean ± SD)**	15.19 ± 2.4	21.3 ± 3.5	22.6 ± 3.6	0.00∗	0.001 **∗**	0.000 **∗**	0.639
**CA (mean ± SD)**	68.12 ± 3.13	65.64 ± 4.13	66.68 ± 2.01	0.493	0.465	0.763	0.867
**DC (mean ± SD)**	60.26 ± 1.25	71.4 ± 0.96	74.96 ± 0.82	0.000∗	0.000 **∗**	0.000 **∗**	0.000 **∗**

NC/GACP: NanoChitosan/Gallic Acid + Calcium Phosphate primer, SD: Standard deviation.

∗: mean significant difference at P < 0.05.

**Fig. 4:**
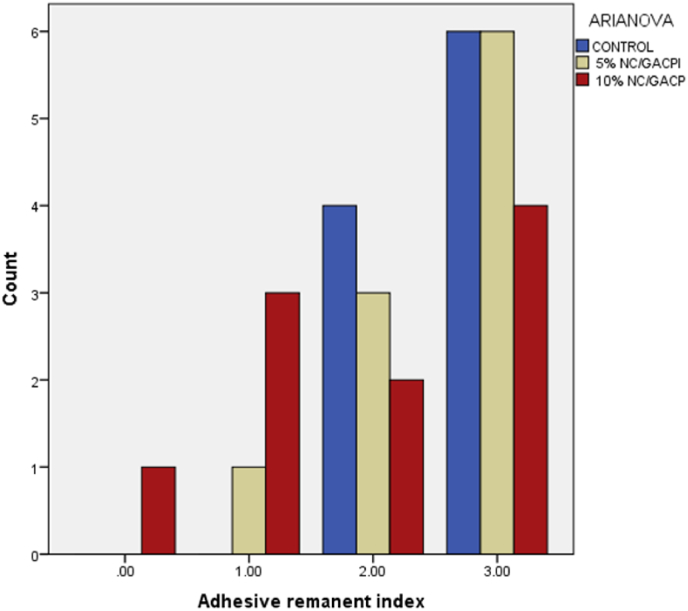
Bar-chart for the distribution of ARI scores among the groups.

The ARI Bar chart in (Fig. 4) showed that the three groups mainly scored 3 (16 specimens), indicating that the adhesives remained in the enamel after bracket debonding, while (9 specimens) scored 2 and the remaining (5 specimens) scored 1 and 0.

The results of wettability in Table 1 showed that the contact angle mean values were 68.12 ± 3.13, 65.64 ± 4.13 and 66.68 ± 2.01 in control, 5 % NC/GACP and 10 % NC/GACP groups respectively with non-significant difference among them (p = 0.493).

For the degree of conversion in Table 1, the highest mean values were 74.96 ± 0.82, followed by 71.4 ± 0.96, and 60.26 ± 1.25, in 10 % NC/GACP, 5 % NC/GACP and control groups respectively with significant differences among them (p = 0.000). The DC was significantly more in 5 % NC/GACP group (P = 0.000) and 10 % NC/GACP group (P = 0.000) than the control group.

The results of the antimicrobial test in Table 2 showed that the experimental disks of both concentrations 5 % and 10 % NC/GACP had an effect against *streptococcus mutans* and *lactobacillus acidophilus* by the presence of clear rings around the disks in the agar plate. The control group had no antibacterial effect (no clear zone around the disk). The clear zone mean values of S. mutans were 18.66 ± 1.1 and 23.42 ± 1.4 while for L. acidophilus were 14.6 ± 0.62 and 19.6 ± 1.05 in 5 % NC/GACP and 10 % NC/GACP groups respectively with significant differences among them (p = 0.000).

**Table 2:** Details of growth inhibition zone against streptococcus mutans and lactobacillus acidophilus among three groups.

Tests	Groups				Post hoc Tukey test∗ p-value		
	Control	5 % NC/GACP	10 % NC/GACP	ANOVA p-value (Sig.)	Control Vs. 5 % NC/GACP	Control Vs. 10 % NC/GACP	5 % NC/GACP Vs. 10 % NC/GACP
**Streptococcus mutans (mean ± SD)**	0.00 ± 0.00	18.66 ± 1.1	23.42 ± 1.4	0.000∗	0.000 **∗**	0.000 **∗**	0.000 **∗**
**Lactobacillus acidophilus (mean ± SD)**	0.00 ± 0.00	14.6 ± 0.62	19.6 ± 1.05	0.000∗	0.000 **∗**	0.000 **∗**	0.000 **∗**

NC/GACP: NanoChitosan/Gallic Acid + Calcium Phosphate primer, SD: Standard deviation.

∗: mean significant difference at P < 0.05.

**Fig. 5:**
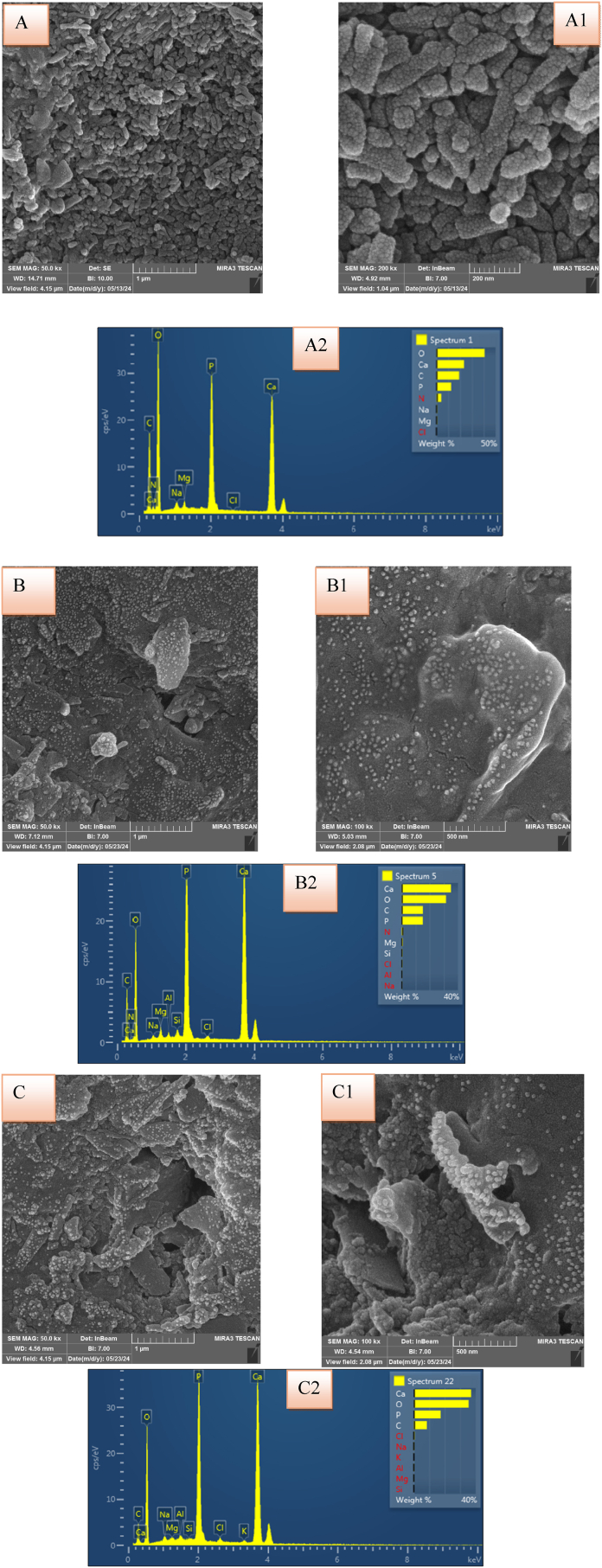
FESEM images of the teeth cross section after remineralization at 50 &100 kx magnifications: A, A1 for control group; B, B1, for 5%NC/GACP; C, C1 for 10%NC/GPCP. A2, B2, C2 showed the EDX analysis for each group.

The FESEM pictures (Fig. 5) showed that the control group had no clear evidence of new mineral precipitation with the presence of gaps between clusters. While a denser and more continuous layer of mineral deposits in the treated groups as the presence of bioactive materials likely enhances mineral ion binding and subsequent precipitation. Furthermore, the gaps or spaces between enamel rods in the control group are wide and poorly filled while in the chitosan loaded with calcium phosphate and gallic acid primer groups, the interrod gaps appeared partially filled, suggesting that the adhesive promotes infiltration of minerals into these spaces and subsequent remineralization. This improves the structural integrity of the enamel by making gaps bridges between rods and reinforcing the framework.

In the EDX analysis (Fig. 5 A2, B2, C2), the primary elements found in enamel were oxygen (O), calcium (Ca), and phosphorus (P) with small amounts of carbon (C), magnesium (Mg), sodium (Na), and chlorate (Cl).

The details of calcium and phosphate concentration among three groups are described in Table 3. The content of calcium (wt %) was 26.42 ± 6.46, 39.76 ± 4.33 and 37.79 ± 3.04 in control, 5 % NC/GACP and 10 % NC/GACP groups respectively. The difference among them was statistically significant (P = 0.002). Compared to control group, the calcium content was significantly more in 5 % NC/GACP (P = 0.002) and 10 % NC/GACP groups (P = 0.008).

**Table-3:** Details of EDX results of calcium weight percent, phosphate weight percent, and Ca/P ratio among three groups.

Elements	Groups				Post hoc Tukey test∗ p-value		
	Control	5 % NC/GACP	10 % NC/GACP	ANOVA p-value (Sig.)	Control Vs. 5 % NC/GACP	Control Vs. 10 % NC/GACP	5 % NC/GACP Vs. 10 % NC/GACP
**Ca wt % (mean ± SD)**	26.42 ± 6.46	39.76 ± 4.33	37.79 ± 3.04	0.002∗	0.002∗	0.008∗	0.798
**P wt % (mean ± SD)**	13.98 ± 2.18	18.68 ± 2.31	17.69 ± 0.78	0.005∗	0.005∗	0.023∗	0.692
**Ca/P (mean ± SD)**	1.87 ± 0.18	2.13 ± 0.13	2.12 ± 0.08	0.017∗	0.029∗	0.03∗	1.00

NC/GACP: NanoChitosan/Gallic Acid + Calcium Phosphate primer, SD: Standard deviation.

∗: mean significant difference at p < 0.05.

The content of phosphate (wt %) was 13.98 ± 2.18, 18.68 ± 2.31 and 17.69 ± 0.78 in control, 5 % NC/GACP and 10 % NC/GACP groups respectively, and the difference among them was statistically significant (P = 0.005). Compared to control group, the phosphate content was significantly more in 5 % NC/GACP (P = 0.005) and 10 % NC/GACP groups (P = 0.023).

The analysis of Ca/P ratio are described in Table 3, the mean values were 1.87 ± 0.18, 2.13 ± 0.13 and 2.12 ± 0.08 in control, 5 % NC/GACP and 10 % NC/GACP groups respectively. The difference among them was statistically significant (P = 0.017). Compared to control group, the Ca/P was significantly more in 5 % NC/GACP (P = 0.029) and 10 % NC/GACP groups (P = 0.03).

## 10 Discussion

Using natural products as antibacterial and remineralizing agents is particularly advantageous. Natural agents often exhibit biocompatibility, reduced cytotoxicity, and a lower risk of adverse effects compared to synthetic chemicals.^21^^,^^22^ Natural polyphenol substances such as gallic acid have shown potent antibacterial activity. Calcium phosphate, a naturally occurring mineral, is highly effective in remineralizing enamel by replenishing essential minerals and enhancing tooth structure. Furthermore, chitosan serves as a drug carrier, enabling the controlled and sustained release of these bioactive materials into demineralized enamel while also exhibiting antibacterial properties.

In this study, the 5 % and 10 % concentrations of nano-chitosan/bioactive materials were selected and added to the orthodontic primer based on previous research. A preliminary pilot study showed that 1 % had no antibacterial effect, while 15 % negatively affected the adhesive's mechanical properties because they reduced the SBS, increased the viscosity and reduced the flowability of the primer. Consequently, 1 % and 15 % were excluded from the study.

The ideal outcome of bracket bonding should ensure a strong attachment capable of withstanding the forces of orthodontic treatment and chewing without dislodging, while also being safe enough to prevent surface damage during debonding at the end of the treatment.^23^

The current study showed that SBS increases by incorporating nano-chitosan loaded with gallic acid and calcium phosphate into orthodontic primer within the acceptable range compared to the control group. These results supported the previous study's finding by Katyal et al.,^24^ who reported that modified orthodontic primer with chitosan has increased the mean value of SBS. Furthermore, the findings agree with those of Shalaby et al.^25^ who found that the GIC modified with 50 % nano-chitosan resulted in nonsignificant increased SBS values as compared to the traditional GIC. The increase in SBS may be attributed to the presence of a chemical link by means of covalent bonds between the universal adhesive and chitosan and resin leading to an increase in the SBS value.^26^

The adhesive remnant index for the SBS varied between scores (2–3) about 53.3 % of the bond failure located between the bracket/adhesive interface, and 36,6 % of the bond failure was cohesively within the adhesive itself, which means that more than half of the orthodontic adhesive remained on the tooth surface after bracket debonding, this finding was in agreement with several previous studies.^27^^,^^28^ A score of 3 for the ARI is advantageous since it reduces the risk of enamel fracture during debonding force.^29^

The bonding of the bracket base is determined by the ability of the primer to wet and adapt to the etched enamel surface, the wettability of the liquid is affected by its contact angle with an inverse relationship between them, suggesting that the wettability getting better at a lower angle.^14^

Our findings were comparable to Katyal et al.,^24^ who demonstrated that the CA of the modified primer was reduced by the addition of chitosan but not statistically significant difference than the control group.

DC was evaluated to determine the amount of the monomers react to form polymers, or the proportion of C=C double bonds that transform into C-C single bonds.^30^ Our study showed the degree of monomer conversion increased significantly and improved adhesive polymerization of both 5 % and 10 % NC/CaGP groups over the control group and this result was comparable to the study of Tanaka et al.,^31^ who added chitosan-loaded with dibasic calcium phosphate fillers which resulted in increasing DC of dental composites.

In contrast, Mahapoka et al.,^32^ study displayed a non-significant decrease in the DC of chitosan whiskers incorporated into dental resin from the control group.

The mean degree of conversion of all the groups was within the clinically normal limit according to the suggestion of Kauppi and Combe^33^; the typical DC ranges from 55 to 75 %. The cause behind improving the degree of monomer conversion in our result is that light curing of the modified primer causes photooxidative degradation of chitosan which leads to the breaking of the polymer chains and produces free radicals which reduce the molecular weight and viscosity of the polymer and increases the polymerization process and results in increasing the DC.^34^

The antibacterial results showed that modified groups had antibacterial effects against both *streptococcus mutans* and *lactobacillus acidophilus* with a significant increase in its activity by increasing the concentration, this agree with Yao et al.,^35^ who found that 20 mg/ml carboxymethyl chitosan (CMC) modified adhesive system, revealed antibacterial action against *S. mutans*. Kikuchi et al.,^36^ showed that composite resin modified by 0.5 % chitosan/dibasic calcium phosphate (DCPA) particles reduced the biofilm formation without affecting the mechanical properties in relation to the control.

Almeshal et al.,^37^ showed that chitosan nanoparticles of different sizes had similar antibacterial effects and showed a reduction in S. mutans by disc agar diffusion method without affecting the SBS of orthodontic adhesive.

The antibacterial activity of chitosan can be linked to its capacity to adhere to the negative charge cell wall and cause blockage of DNA replication and cell destruction, another reason for its antibacterial properties due to its functions as an adhesive agent, attaching itself to the toxins-producing organism, preventing it from growing.^24^

In another study, Elsharkawy et al.,^38^ reported that the addition of different concentrations of gallic acid to the glass ionomer cement showed a significant antibacterial turbidity reduction when compared to the control group. Their antibacterial properties are due to the ability of GA to permanently alter the bacterial cell membranes, this may lead to changes in the cell wall charge, localized rupture, or pore development that results in the subsequent leakage of major intracellular components.^39^

The findings of FESEM and EDX indicate that both 5 and 10 % NC/GACP groups can remineralize the demineralized enamel as greater calcium, phosphate, and Ca/P ratio than the control group. Our results align with those of Mohamed & Ashraf,^40^ who found that EDX analysis revealed a higher percentage of calcium (Ca) and phosphorus (P), as well as an increased Ca/P ratio, in the phosphorylated chitosan group compared to the silver diamine fluoride and sodium fluoride varnish groups. Similarly, Aboayana et al.,^41^ reported that the chitosan nanoparticle group exhibited higher Ca and P percentages and a higher Ca/P ratio compared to the silver nanoparticle and control groups.

Our findings showed that 5 % NC/GACP primer group showed nonsignificant greater Ca, P weight percentages and Ca/P ratio than the 10 % NC/GACP primer groups. This result agrees with Ashour et al.,^42^ who reported no significant difference in Ca/P ratio between 2.5 % and 5 % phosphorylated chitosan/amorphous calcium phosphate nanocomplexes groups.

A possible explanation for this might be that chitosan functions as a scaffold in the biomineralization process, guiding the formation of mineral crystallites through molecular interactions between the polymer and minerals. In this study, higher concentrations led to saturation of subsurface lesions. However, higher concentrations of remineralizing agents increase chitosan viscosity, which may inversely affect its permeability and result in less Ca and P release.^42^

From all these studies we can conclude that the ability of chitosan to induce enamel remineralization is due to the fact that chitosan has an adhesive property so in acidic media, chitosan's amino groups become protonated, resulting in positively charged molecules that adhere to negatively charged surfaces tooth enamel.^43^ Also the structure of chitosan acts as a drug delivery system because it has specialized active regions that combine with various bioactive materials to release ions required in remineralization.^44^

Tang et al.^45^ proposed that when gallic acid was absent, crystals did not organize, while in the existence of gallic acid, crystals changed to form spherules. The crystal's size decreased as the concentration of gallic acid increased. All these findings indicated that gallic acid has a role in the morphology and growth of HAP crystals, which is considered the key mechanism for gallic acid in remineralization. However, more similar studies are required to confirm the present findings. This study has certain limitations, including its in vitro design conducted under controlled laboratory conditions, which may not accurately reflect the complexity of the oral environment. Additionally, the remineralization assessment was limited to a fixed period of four months. Further studies are needed to evaluate its long-term effectiveness and its impact on a wider range of bacteria, including oral biofilm and other cariogenic pathogens.

It would also be valuable to explore the potential of incorporating chitosan/gallic acid and calcium phosphate into other dental products, such as toothpaste, varnishes, and other preventive applications.

## 11 Conclusions

1. The 10 % NC/GACP improved the SBS, DC, and antibacterial activity more than 5 % NC/GACP and control groups.
2. The 5 % NC/GACP exhibited a higher calcium and phosphate weight percentage, as well as a greater Ca/P ratio than the 10 % NC/GACP and control groups, suggesting its potential for remineralization.
3. The Adhesive Remnant Index (ARI) scores were mostly 3, which is advantageous in minimizing enamel fracture during debonding.
4. No significant differences were observed in contact angle measurements among the groups, indicating that the modified adhesive did not affect the wettability of the primer.
5. The modified primer is considered a novel adhesive for the treatment of white spot lesions (WSLs) due to its antibacterial effect against cariogenic bacteria and its ability to remineralize the demineralized enamel.

## Ethical clearance

This research has been approved by the ethical committee at the College of Dentistry/University of Mosul under the no (UoM.Dent. 23/24) on May 02, 2023.

### Patient's/Guardian's consent

This research did not involve the use of patients' clinical images or radiographs. The study only utilized teeth collected from patients at specialized centers who had their teeth extracted for orthodontic treatment purposes.

## Financial support and sponsorship

Nil.

## Declaration of competing interest

The authors declare that they have no known competing financial interests or personal relationships that could have appeared to influence the work reported in this paper.
